# A comparative study of two different clear aligner systems

**DOI:** 10.1186/s40510-014-0031-3

**Published:** 2014-05-02

**Authors:** Federica Ercoli, Michele Tepedino, Vincenzo Parziale, Cesare Luzi

**Affiliations:** 1 Department of Biotechnological and Applied Clinical Sciences, University of L’Aquila, Via Ospedale S. Salvatore, 6, Coppito L’Aquila 67100, Italy; 2 Rome, Italy

**Keywords:** Clear aligners, Invisible appliances, Nuvola®, Fantasmino®

## Abstract

**Background:**

This study aims to compare the ‘Nuvola®’ system with ‘Fantasmino®’ system, examine their material properties, and define the indications for use of the aligners.

**Methods:**

Two groups of patients were selected and were respectively treated with Nuvola® aligner and Fantasmino® system.

**Results:**

The goal of treatment has been achieved with the two systems.

**Conclusions:**

The two types of aligners have shown differences during the treatment. Fantasmino® system has elastic properties of high performance, but its size does not encourage compliance throughout the day. Nuvola® system determines good tooth movement and its size facilitates the patient’s collaboration. In both aligner systems, difficulties were found in the correction of torque information and rotations.

## Background

Invisible orthodontic techniques, such as clear aligners and lingual appliances, have deeply changed orthodontics, allowing patients with particular professional and social needs [1,2] to undertake treatment. Requests for invisible appliances come generally from adult patients [3] who have contact with the public [4], professionals of the show business, adults who experience late crowding or relapse after a conventional orthodontic treatment, professional athletes, and also adolescents [5]. Aligners can be made of different polymers [6] and have the advantage of being removable devices. The aim of this study is to compare two systems of aligners: Nuvola® and Fantasmino®.

## Methods

The study group was selected according to the following inclusion criteria:

 Class I, II [7], and III malocclusions [8]

 Mild and moderate dental crowding [9] (assessed through the Little Irregularity Index [10], with an average value of 5.07 mm)

 Pre-prosthetic orthodontic treatment

 No need for extractions

 No need for orthognatic surgery

**Figure 1:**
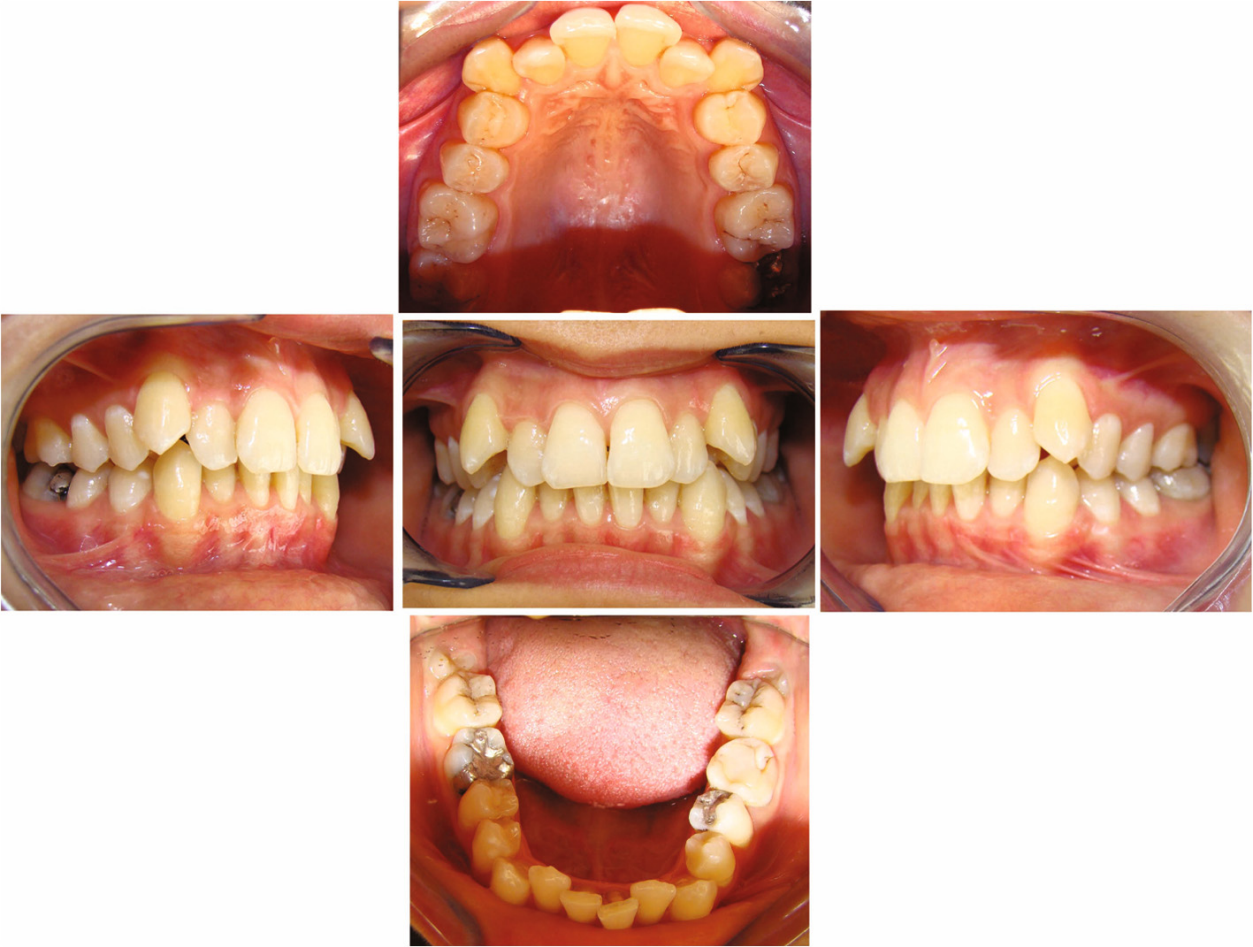
**Fantasmino® system, pre-treatment photographs of a case.** The front, right side, left side, upper, and lower occlusal patient's photos.

**Figure 2:**
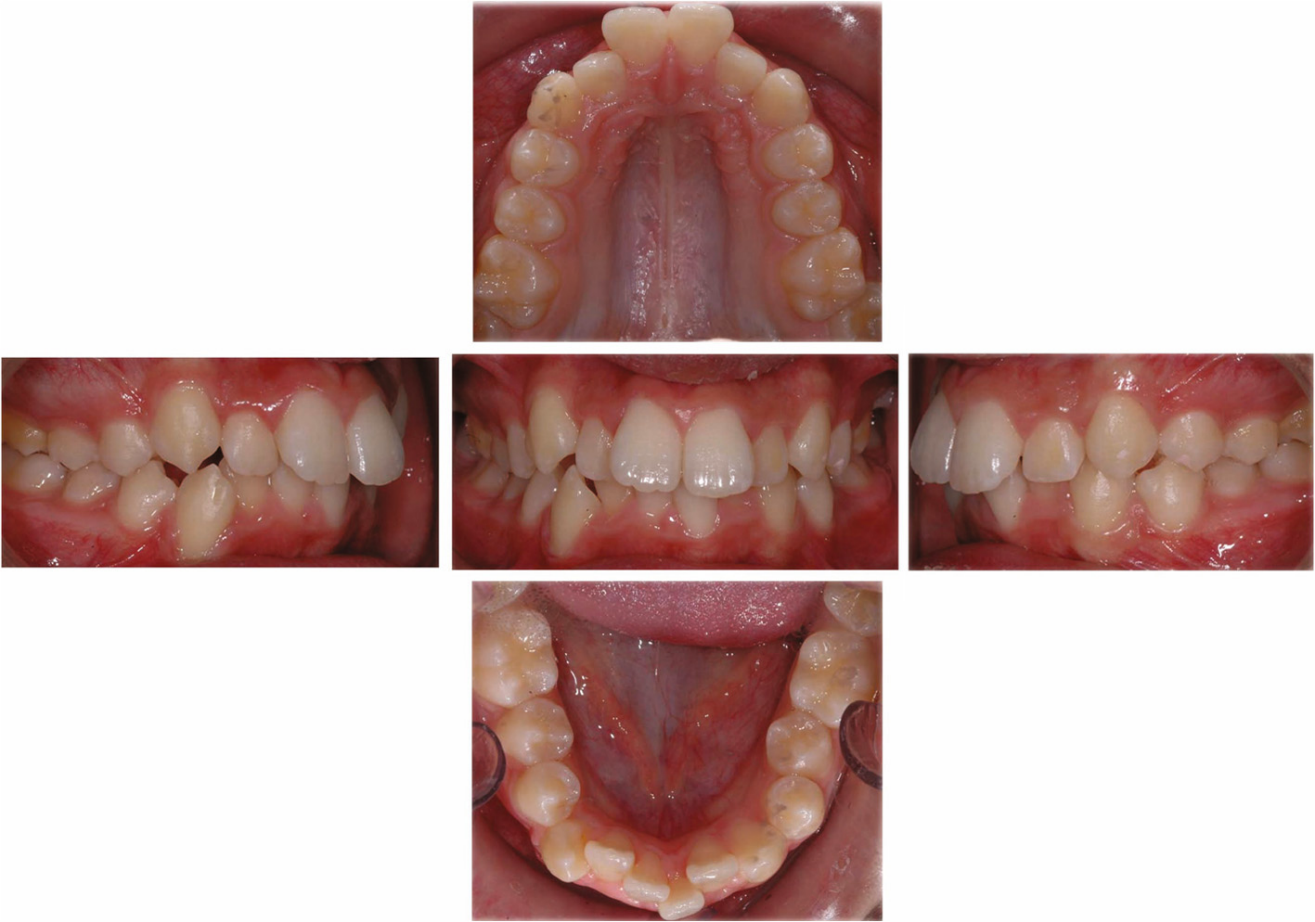
**Nuvola® system, pre-treatment photographs of a case.** Front, right side, left side, upper and lower occlusal patient's photos.

Twenty patients responding to the inclusion criteria were selected. The sample was composed of 12 females and 8 males, ranging in age from 16 to 45 years (mean 31.7 ± 8.7 years). An operator not involved in the study divided the patients into two groups: group A (seven females, three males) was treated with the Fantasmino® system (Figure 1), and group B (five females, five males) was treated with the Nuvola® system (Figure 2). For both systems, the number of aligners utilized ranged from 8 to 14 (mean number 10.8), and the mean treatment time was 5.8 months (about 15 days for each aligner).

The two groups were compared by patient’s satisfaction, improvement of the irregularity index (Little’s Irregularity Index is defined as the summed displacement of adjacent anatomical contact point of the six mandibular anterior teeth [11]), speech impairment, and mean wear time. Patients were asked to wear the appliances for a different time according to the used aligner system: 14 h per day for group A and 22 h per day for group B.

## Results

**Figure 3:**
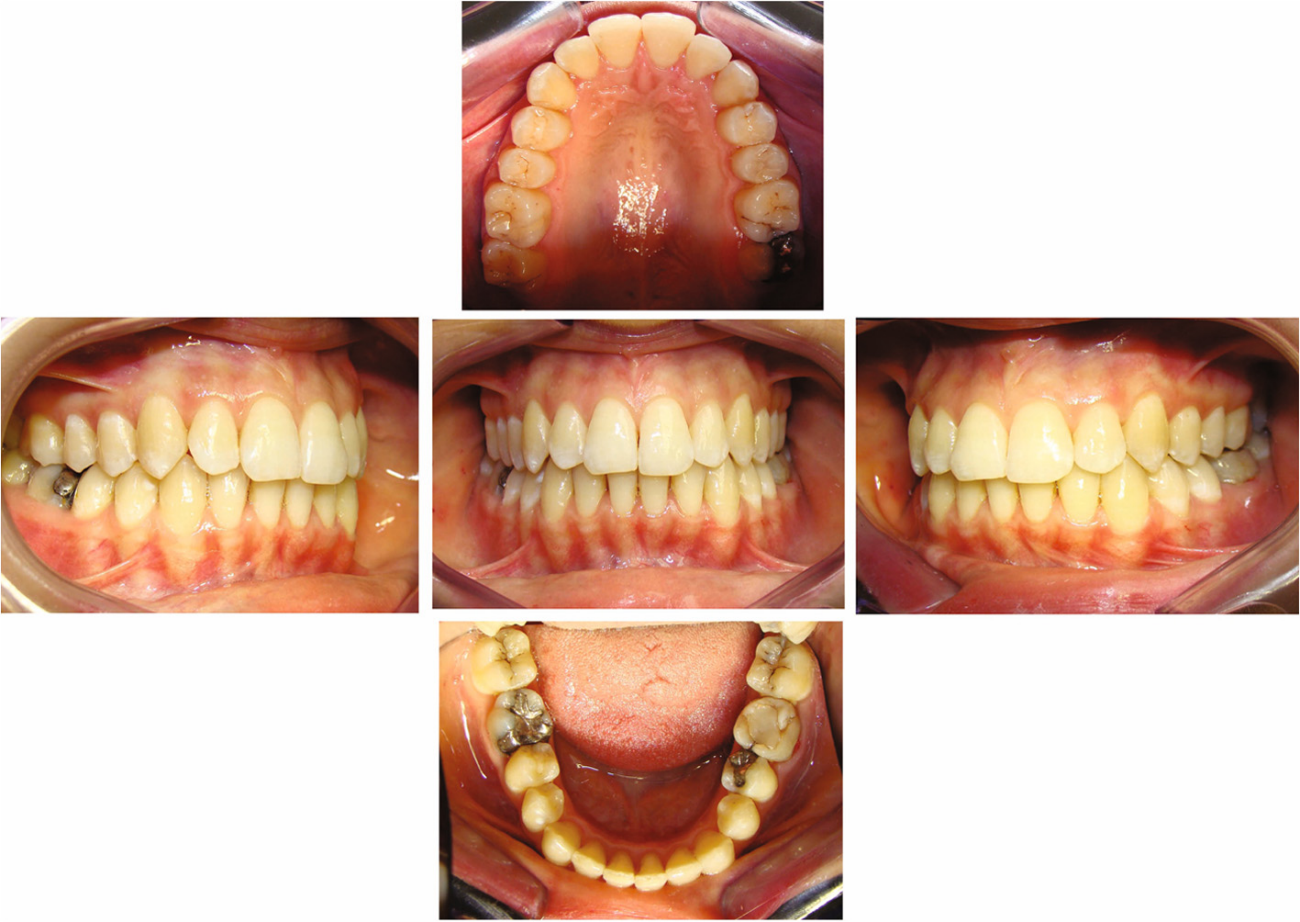
**Fantasmino® system, post-treatment records of case shown in Figure**1**.** Front, right side, left side, upper and lower occlusal patient's photos.

**Figure 4:**
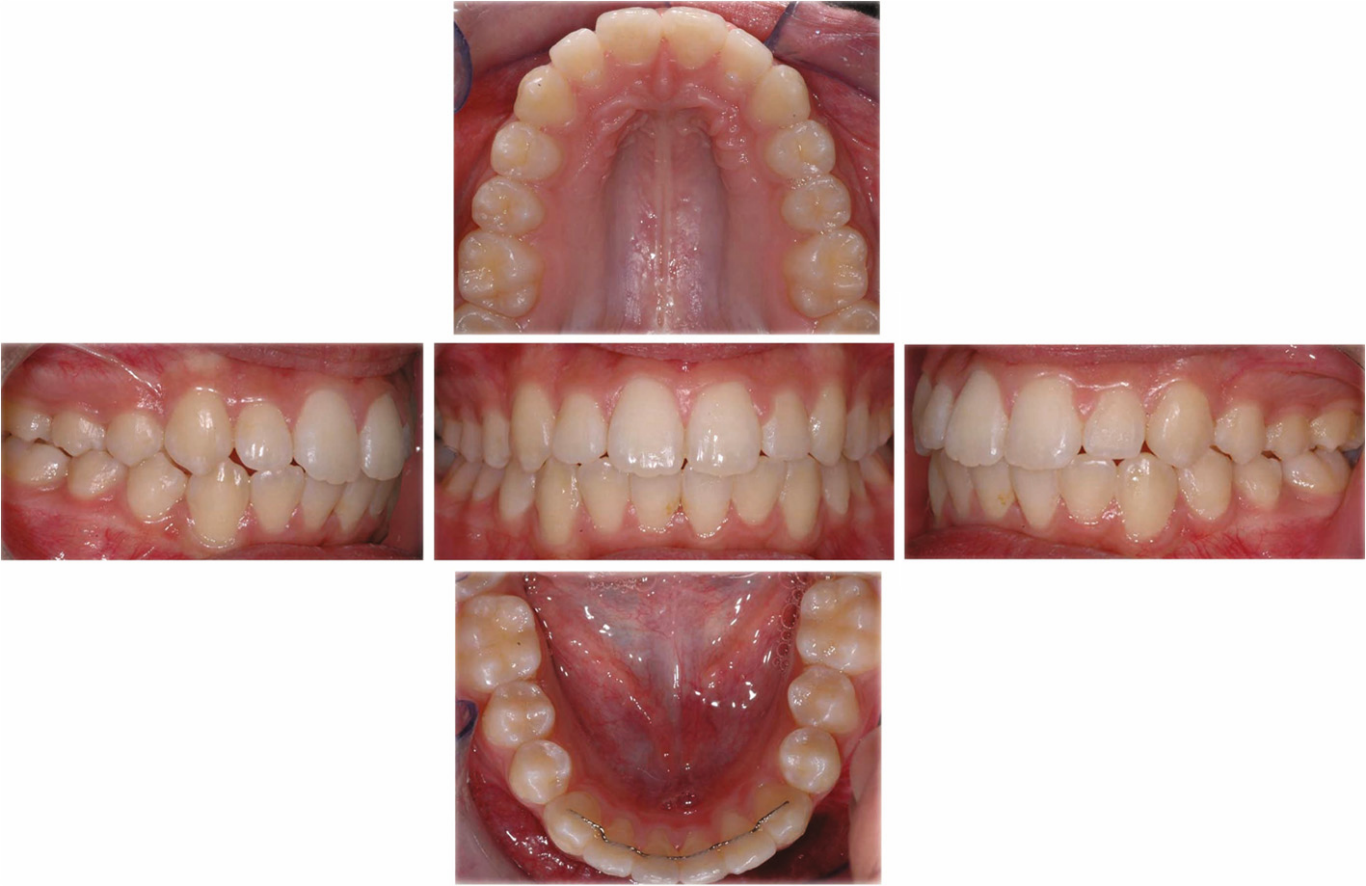
**Nuvola® system, post-treatment records of case shown in Figure**2**.** Front, right side, left side, upper and lower occlusal patient's photos.

The two systems showed no difference in patient’s satisfaction, improvement of the irregularity index, speech impairment, and mean wear time. One of the male patients assigned to group B was excluded from the study due to poor compliance. Patients from both groups referred a high level of satisfaction at the end of treatment. Dental alignment and arch coordination at the end of treatment were comparable to the predicted result during the planning phase with both system softwares (Figures 3 and 4).

At the beginning of treatment, the average index value was 5.08 mm for group A and 4.97 mm for group B. Following alignment, both systems have shown a reduction of the average index value of 0.69 mm for group A and 0.64 mm for group B (Tables 1 and 2).

**Table 1:** Little’s irregularity index values pre- and post-treatment for patients treated with Fantasmino® system (group A)

**Patients using Fantasmino®**	**Pre-treatment values(mm)**	**Post-treatment values(mm)**
1	3.65	0.30
2	5.60	0.90
3	4.00	0.50
4	5.91	0.90
5	3.20	0.30
6	5.73	0.80
7	6.51	1.00
8	3.40	0.40
9	6.00	0.80
10	6.86	1.00
Mean values	5.08 ± 1.37	0.69 ± 0.28

**Table 2:** Little’s irregularity index values pre- and post-treatment for patients treated with Nuvola® system (group B)

**Patients using Nuvola®**	**Pre-treatment values(mm)**	**Post-treatment values(mm)**
1	3.20	0.30
2	5.64	0.50
3	6.30	0.90
4	3.70	0.40
5	3.50	0.40
6	6.40	0.90
7	6.70	1.00
8	5.20	0.80
9	4.10	0.50
Mean values	4.97 ± 1.37	0.63 ± 0.26

Group A patients showed difficulties pronouncing certain phonemes (t\d\s\z\ts\dz\l\r), which decreased during treatment. Group B did not show this kind of impairment. All patients selected showed good compliance. Patients of group A expressed appreciation for the reduced wear time and the possibility to choose when to wear the aligners.

## Discussion

The two systems use aligners made of different polymers [12-14]. Fantasmino® aligners are made of poly-vinyl chloride (PVC), a material with elastic characteristics following a plastic deformation when exposed to moderate loads. This characteristic allows reducing the optimal wear time to 14 h per day: the deformations subdued by the aligner when worn generate a force that is transferred to the teeth. The thickness of the PVC aligners varies with the desired type of tooth movement but never exceeds 1 mm.

Nuvola® aligners are made of polyethylene terephthalate glycol (PETG), a light, resistant, and very clear material. It is resistant to time and wear, and its elasticity allows for a gradual tooth movement. PETG aligners have a thickness that changes throughout the different treatment phases: 0.75 mm at the beginning of treatment, 0.85 mm during the intermediate phase, and 1 mm at the end of treatment. This system requires an optimal wear time of 22 h.

Both systems can take advantage of auxiliaries to facilitate dental movement, such as composite attachments [15] bonded to the buccal or lingual tooth surfaces. Attachments can have different shapes and sizes, depending of the kind of tooth movement required. It is possible to use an etching jig, with holes corresponding to the desired position of the attachments, in order to avoid undesired demineralization of an excessive portion of tooth enamel. The Fantasmino® system allows the use springs in Beta Titanium or Australian wire (AJ Wilcock, Whittlesea, Victoria, Australia), to enhance rotation and tipping or uprighting of teeth, hooks or vestibular archwires.

The construction phases of both aligner systems are the following:

 Scanning of plaster casts

 Conversion of the scans into Stereo Lithography Interface Format or Standard Triangulation Language (STL)

 Virtual set-up of orthodontic tooth movements

 Printing of the set-up models through rapid prototyping

 Thermoforming of the aligners (PVC or PETG)

Laser scanners (structured light scanners) are used by both systems to acquire images of the plaster casts. Nuvola® system uses a dedicated software, NUVOLA CAD 3D (a CAD plug-in from Rhinoceros, Robert McNeel & Associates, Rome, Italy), while Fantasmino® system uses a software CAD created by Ortolab Pompei (Pompei Napoli, Italy). The images acquired need to be converted into STL format, a file format used in CAD stereolithography. The STL format represents a solid (in this case the patient’s plaster casts) through a mesh of triangles in a 3D environment. This conversion is necessary to rapid prototyping [16], which is an additional technique where a resinous material is apposed layer by layer. Starting from the images of the patient’s dental casts, processed as explained above, the desired orthodontic movements are planned and divided into subsequent phases. For each phase, a model of the virtual set-up is printed through rapid prototyping. These models are used to create a series of dental aligners, thanks to a thermoforming process.

## Conclusions

Although all clear aligner systems have shown to have evident biomechanical limits [17,18], barely producing bodily movement and expressing torque, both clear aligner systems in this study showed good treatment efficiency. To obtain a good dental alignment and arch coordination, it is mandatory to make a correct diagnosis and to choose treatment objective achievable with the limited biomechanics offered by clear aligners. Another key factor is to investigate the patient’s expectations and social and professional needs in order to choose the most appropriate appliance.

## Competing interests

The authors declare that they have no competing interests.

## Authors’ contributions

FE has drafted the manuscript and treated three clinical cases with Fantasmino® system and two cases with Nuvola® system. MT has conducted the measurements on the models and treated two cases with Fantasmino® system and two cases with Nuvola® system. VP has drafted bibliographic information and treated one case with Fantasmino® system and one case with Nuvola® system. CL has treated four cases with Fantasmino ® system and four cases with Nuvola® system. All authors have read and approved the final manuscript.
